# Editorial: Novel biomarkers for predicting response to cancer immunotherapy

**DOI:** 10.3389/fimmu.2023.1179913

**Published:** 2023-05-11

**Authors:** Shuchen Huangfu, Jinghua Pan

**Affiliations:** Department of General Surgery, the First Affiliated Hospital of Jinan University, Guangzhou, China

**Keywords:** immune markers, cancer, ICI, immune response, tumor mutation burden

Immune response refers to the process by which immune cells recognize antigen molecules, activate, proliferate, differentiate and produce immune substances after being stimulated by antigens. It includes a series of physiological reactions such as antigen presentation, lymphocyte activation, immune molecule formation and immune effect. Immune response is the focus of tumor treatment and plays an important role in the research and clinical application of a variety of malignant tumors. This project summarizes the role of immune response markers in cancer therapy. The topic consisted of eight articles, including one review article and seven original research articles, contributed by multiple authors in the fields of tumor immunology and therapeutics. Our goal is to reveal the role of novel markers of tumor immune response in tumor therapy.

As a new treatment method, immunotherapy has gradually become the fourth treatment method in addition to surgery, chemotherapy and radiotherapy. However, due to the limited response rate of immunotherapy, in order to further improve the efficiency of immunotherapy, it is urgent to explore new targets of tumor immune response to achieve the purpose of precise treatment. As the only biomarker that has been confirmed to have predictive function in prospective clinical trials, PD-L1 has been approved by FDA as an immune checkpoint inhibitor (ICI) and is widely used in clinical practice. The degree and distribution of TILs in tumor and its microenvironment can be used as an important predictor of ICI response. Chong Sun et al. conclude that antibody-based PD-1-PD-L1 inhibitors can induce durable tumor responses in patients with a variety of advanced cancers (1).The degree and distribution of TILs in tumor and its microenvironment can be used as an important predictor of ICI response. Savas P et al. found that CD8^+^ TRM cells contribute to breast cancer immune surveillance and are a key target of immune checkpoint inhibition regulation (2). tumor mutation burden is the focus of research on tumor immune response markers in recent years. The higher the TMB of the tumor, the higher the immunogenicity of the tumor. Using systemic treatment with the Axl inhibitor bemcentinib in combination with PD-1 checkpoint blocker treatment, Huiyu Li et al. found that Axl achieves anti-PD-1-mediated growth control of STK11/LKB1 mutant NSCLC by expanding CD8^+^ T cells, the main executor of TCF1^+^PD-1^+^ (3).By analyzing the immunophenotype of 188 melanoma patients treated with ICB, Shen R et al. found that LAG-3 expression in peripheral blood cells could identify patients with poor prognosis after ICB, and this research result has a guiding role for immunotherapy of LAG^+^ immune melanoma patients (4).

Gene-expression markers are widely and equally comprehensive in assessing tumor response to ICI therapy. Jia K et al. showed that claudin-18 (CLDN18.2) positive gastric cancer (GC) has unique immune microenvironment characteristics, which makes CLDN18.2 positive GC have relatively fewer CD8/CD4 T cells expressing PD-1/PD-L1. This results in poor survival of patients receiving anti-PD-1/anti-PD-L1 therapy, indicating that CLDN18.2 may be a promising new therapeutic target (5). In addition, Shuai Wang et al. found that CD47 blockade significantly enhanced the ability of CD103^+^ DCs to uptake tumor DNA in the HCC microenvironment, thereby stimulating the cGAS-STING pathway and promoting the infiltration and activation of NK cells in HCC, suggesting the role of CD47 blockade in HCC treatment (6). Similarly, results of a phase I trial of an anti-CD47 monoclonal antibody (Hu5F9-G) conducted by Branimir I Sikic et al. in patients with solid tumors and Hodgkin lymphoma showed that Hu5F9-G was well tolerated in patients with solid tumors and lymphoma when administered using priming and maintenance dose regimens (7).

As a class of signaling cytokines, chemokines participate in the important process of tumor immune response by interacting with receptors to regulate immune infiltration and activation of host immune response. By analyzing the chemokine landscape and immune infiltration in metastatic melanoma samples using protein markers and RNA transcript imaging based on multiplex mass spectrometry flow cytometry, Tobias Hoch et al. found that CXCL9 and CXCL10-enriched tumor microenvironment (TME) contributes to the generation of a “hot” tumor microenvironment, It has a predictive effect on OS of melanoma patients (8).

In summary, the project “Novel markers of tumor immune response” highlights the important role of the exploration of new markers of tumor immune response in the prediction of tumor ICI treatment response, precision immune therapy, and prognosis of immunotherapy. This research direction provides great prospects for tumor immunotherapy.

## Author contributions

SH drafted the manuscript. JP revised the manuscript. All authors listed have made a substantial, direct, and intellectual contribution to the work, and approved it for publication.
